# Oxidative Stress-Tolerant Stem Cells from Human Exfoliated Deciduous Teeth Decrease Hydrogen Peroxide-Induced Damage in Organotypic Brain Slice Cultures from Adult Mice

**DOI:** 10.3390/ijms20081858

**Published:** 2019-04-15

**Authors:** Li Xiao, Chikako Saiki, Hisashi Okamura

**Affiliations:** 1 Department of Pharmacology, School of Life Dentistry at Tokyo, The Nippon Dental University, 1-9-20 Fujimi, Chiyoda-ku, Tokyo 102-0071, Japan; 2 Department of Physiology, School of Life Dentistry at Tokyo, The Nippon Dental University, 1-9-20 Fujimi, Chiyoda-ku, Tokyo 102-0071, Japan; chikako@tky.ndu.ac.jp; 3 Department of Oral and Maxillofacial Surgery, The Nippon Dental University Hospital, 2-3-16 Fujimi, Chiyoda-ku, Tokyo 102-8158, Japan; hisashi124@gmail.com

**Keywords:** oxidative stress-tolerant stem cells, brain damage, brain slice culture, stem cells from human exfoliated deciduous teeth, neuronal protection

## Abstract

Oxidative stress causes severe tissue injury of the central nervous system in ischemic brain damage (IBD), traumatic brain injury (TBI) and neurodegenerative disorders. In this study, we used hydrogen peroxide (H_2_O_2_) to induce oxidative stress in organotypic brain slice cultures (OBSCs), and investigated the protective effects of oxidative stress-tolerant (OST) stem cells harvested from human exfoliated deciduous teeth (SHED) which were co-cultivated with OBSCs. Using presto blue assay and immunostaining, we demonstrated that both normal SHED and OST-SHED could prevent H_2_O_2_-induced cell death, and increase the numbers of mature neuron and neuronal progenitors in the hippocampus of OBSCs. During co-cultivation, OST-SHED, but not normal SHED, exhibited neuronal cell morphology and expressed neuronal markers. Results from ELISA showed that both normal SHED and OST-SHED significantly decreased oxidative DNA damage in H_2_O_2_-treated OBSCs. SHED could also produce neurotrophic factor BDNF (brain derived neurotrophic factor) and promoted the production of IL-6 in OBSCs. Although OST-SHED had lower cell viability, the neuronal protection of OST-SHED was significantly superior to that of normal SHED. Our findings suggest that SHED, especially OST-SHED, could prevent oxidative stress induced brain damage. OST-SHED can be explored as a new therapeutic tool for IBD, TBI and neurodegenerative disorders.

## 1. Introduction

Brain tissue is highly sensitive to reactive oxygen species (ROS)-induced oxidative stress because it has a high rate of oxygen consumption, high lipid-rich contents, high concentrations of transition metals such as iron and copper, and low antioxidant capacity [1,2]. ROS include superoxide anion (O_2_^−^), hydrogen peroxide (H_2_O_2_), and hydroxyl radical (HO·). ROS at physiological level play essential roles in the cellular signaling pathway [3]. However, in aged or injured brains, excessive amounts of ROS are generated [4,5]. When ROS overwhelm the cellular antioxidant defense system, oxidative stress occurs [6]. Oxidative stress is associated with cellular damage seen in ischemic brain damage (such as strokes), traumatic brain injury (TBI) and neurodegenerative disorders (including Parkinson’s and Alzheimer’s disease). Yet, the use of antioxidants to treat the above-mentioned diseases has not shown hopeful clinical outcomes [7,8,9].

Recently, stem cell-based therapy has been considered as a promising strategy for neurodegenerative diseases, stroke and TBI [10,11]. Transplantation of human embryonic stem (ES) cells, induced pluripotent stem (iPS) cells and mesenchymal stem cells (MSCs) could improve the functionality of the CNS and provide neurotrophic support to local tissue in animal models of neurodegenerative diseases and stroke [10]. However, transplanted human stem cells usually suffer massive cell death due to oxidative stress in the local environment. Thus, oxidative stress-tolerant stem cells are considered desirable for stem cell-based therapy [12].

Stem cells from human exfoliated deciduous teeth (SHED) are isolated from the remnant pulp of exfoliated deciduous teeth. SHED are respected as high-quality human postnatal stem cells due to their high proliferation and capacity for multilineage differentiation [13]. SHED (as well as SHED from intact cryopreserved deciduous teeth) could differentiate into various cell types, including odontoblasts, osteoblasts, chondrocytes and adipocytes [14,15]. Under neural differentiation SHED could express neuronal markers, such as βIII-tubulin, GAD (Glutamate decarboxylase), and NeuN. When SHED were transplanted into the dentate gyrus (DG) of the hippocampus of immunocompromised mice, some of them settled in the DG region and expressed neuronal marker NFM (neurofilament medium chain) [13]. This evidence suggests that SHED have the potential for neuronal regeneration. However, to date no literature has reported the interaction between SHED and oxidative stress-damaged brain tissue.

Organotypic brain slice cultures (OBSCs) have been used as tools for testing the responses of brain tissue to oxidative stress [16], and investigating the cellular and molecular bases of neurodegenerative diseases in vitro [17,18]. In our previous study, we invented a novel method to maintain adult mouse hippocampal slices in vitro for long-term culture by using a matrigel-coated culture insert. We also observed that human adult dental pulp cells could produce neurotrophic factors and increase cell viability in co-cultivated hippocampus slices [19]. In the present study, we discovered a method to obtain oxidative stress-tolerant SHED (OST-SHED) and investigated the protective effects of OST-SHED on H_2_O_2_-induced oxidative stress injuries in OBSCs.

## 2. Results

### 2.1. Establishment of Oxidative Stress-Tolerant SHED (OST-SHED)

**Figure 1:**
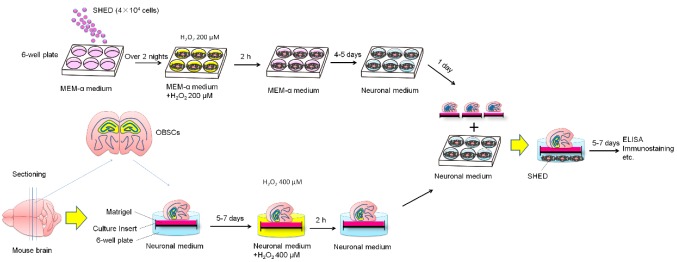
Experimental setup and cultivation schedule.

**Figure 2:**
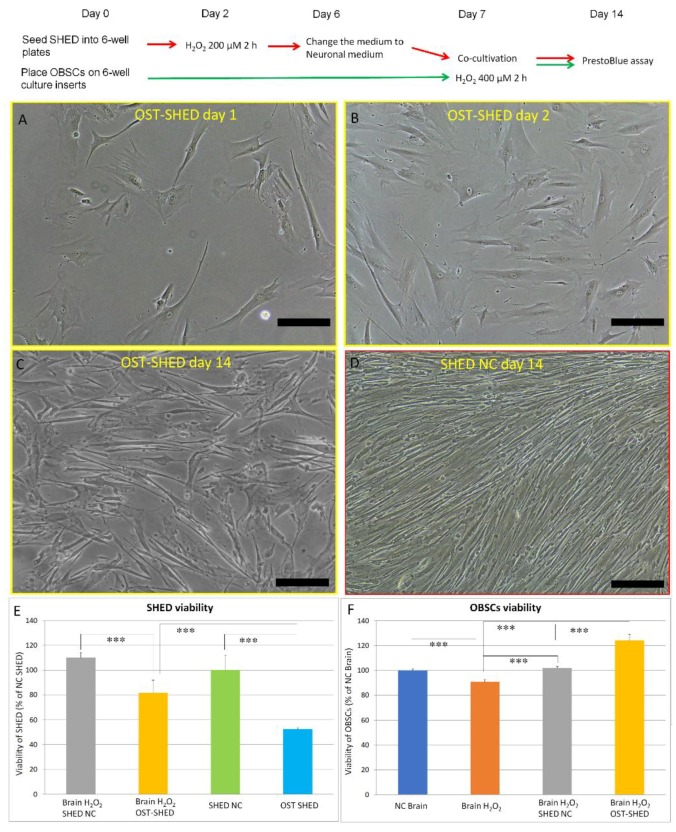
Cell viability of OBSCs and SHED at the end of cultivation. (**A**–**C**), Morphology of OST-SHED. (**A**), One day after H_2_O_2_ treatment; (**B**), Two days after H_2_O_2_ treatment; (**C**), Two weeks after H_2_O_2_ treatment. (**D**), Morphology of normal SHED (negative control, SHED NC), cells were cultivated under the same conditions without H_2_O_2_ treatment. Scale bar = 50 μm. (**E**,**F**), At the end of cultivation, the culture inserts (which containing OBSCs) were transferred into new 6-well plates and followed by presto blue assay. Co-cultivated SHED and solo-cultivated SHED were also followed by presto blue assay. Data are presented as mean ± SD. *** *p* < 0.001. SHED NC (normal SHED), SHED without H_2_O_2_ treatment, OST-SHED, SHED with H_2_O_2_ treatment, NC Brain, OBSCs solo-cultivated without H_2_O_2_ treatment, Brain H_2_O_2_, OBSCs solo-cultivated with H_2_O_2_ treatment, Brain H_2_O_2_ SHED NC, OBSCs treated with H_2_O_2_ and co-cultivated with normal SHED, Brain H_2_O_2_ OST-SHED, OBSCs treated with H_2_O_2_ and co-cultivated with OST-SHED, (The abbreviations are also applied in the rests).

To obtain OST-SHED, we treated SHED with 200 μM H_2_O_2_ for 2 h. After rinsing out H_2_O_2_, the cells were further cultivated for about 4–5 days (Figure 1). We noticed that after H_2_O_2_ treatment, SHED presented larger cell bodies and had more dendrites. However, H_2_O_2_-treated cells did not stop proliferation (Figure 2A–C). At the end of cultivation (solo-cultivation, 7 days in regular medium and 7 days in neuronal medium), the viability of H_2_O_2_-treated cells were about 50% of the control cells (Figure 2E). This data suggested that H_2_O_2_-treated SHED are tolerant to H_2_O_2_-induced oxidative stress. Therefore, we named these cells OST-SHED.

At day 0, SHED (4 × 10^4^) were seeded into 6-well plates. After two days of cultivation, SHED were treated with H_2_O_2_ 200 μM for two h. SHED were further cultivated for 4–5 days, and then the medium was changed to neuronal medium for one day. At the same time, mice brains were sectioned to slices with 200 μm thickness. The brain slices (OBSCs) were cultivated on matrigel-coated culture inserts in 6-well plates. 5–7 days (The culture time depends on if there are any migrated cells near the edge of OBSCs) later, OBSCs were treated with H_2_O_2_ 400 μM for two h. OBSCs were then transferred into the SHED culture wells and co-cultivated with SHED for another 5–7 days. Medium was changed two times a week. At the end of cultivation, culture supernatant and brain tissue were harvest for ELISA, Immunostaining, etc.

### 2.2. Effect of Normal SHED and OST-SHED on Viability of Co-cultivated OBSCs

We compared the protective effects of both OST-SHED and normal SHED in H_2_O_2_-treated OBSCs. We examined cell viability in both OBSCs and SHED by using presto blue assay. As shown in Figure 2F, H_2_O_2_ (400 μM) treatment significantly decreased cell viability in solo-cultivated OBSCs. Both normal SHED and OST-SHED markedly increased cell viability in co-cultivated OBSCs (*p* < 0.001). Interestingly, although cell viability in OST-SHED was much lower than that in normal SHED in both solo- and co-culture (Figure 2E) (*p* < 0.001), the viability of co-cultivated OBSCs are significantly higher in the OST-SHED group than that in the normal SHED group (Figure 2F) (*p* < 0.001). Moreover, cell viability in co-cultivated OST-SHED was significantly higher than that in solo-cultivated OST-SHED, suggesting that OBSCs could also conversely stimulate the proliferation of OST-SHED.

### 2.3. Effect of SHED on H_2_O_2_-Induced Mature Neuron Death and Supression of Neuronal Generation

**Figure 3:**
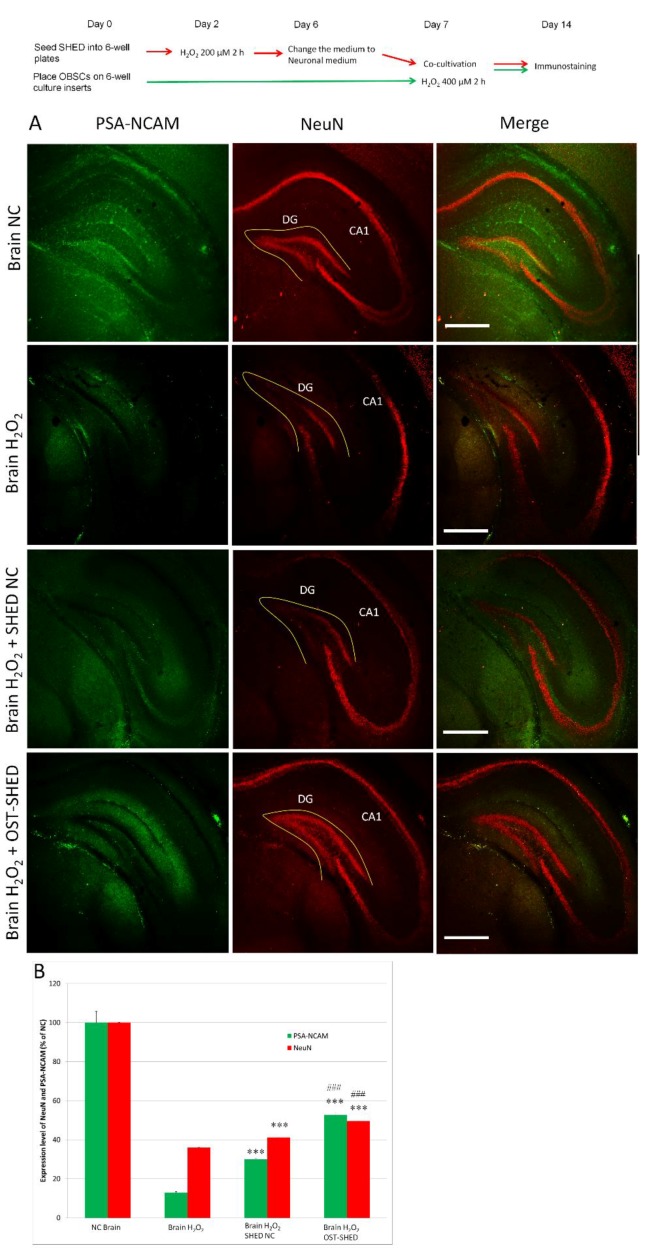
Expression of PSA-NCAM and NeuN in OBSCs. At the end of cultivation (day 14), OBSCs were fixed with 4% paraformaldehyde and followed by immunostaining. (**A**), Typical images of the expression of PSA-NCAM (neuronal regeneration maker) (green) and NeuN (mature neuron marker) (red) in the hippocampus region in OBSCs. DG, dentate gyrus; (**B**), Expression level of PSA-NCAM and NeuN in OBSCs were analyzed with Image J software by quantifying the fluorescence density. Data are presented as mean ± SD. *** *p* < 0.001 v.s. Brain H_2_O_2_, ### *p* < 0.001 v.s. Brain H_2_O_2_ SHED NC.

Oxidative stress-induced death of mature neurons and suppression of neuronal generation are the major causes leading to CNS dysfunction. Hence, we checked the expressions of NeuN (mature neuron marker) and PSA-NCAM [polysialylated-neural cell adhesion molecule, a marker for developing and migrating neurons (neuronal progenitors)] in OBSCs. Figure 3 shows that at 7 days after H_2_O_2_ treatment, anti-NeuN antibody positive cells and anti-PSA-NCAM antibody positive cells were greatly reduced in solo-cultivated OBSCs. Both normal and OST-SHED significantly increased the number of NeuN positive and PSA-NCAM positive cells in the hippocampus region in co-cultivated OBSCs. Moreover, OST-SHED showed remarkable superiority to normal SHED (*p* < 0.001).

### 2.4. Spontaneous Neuronal Differentiation in Normal and OST-SHED Co-cultivated with OBSCs

**Figure 4:**
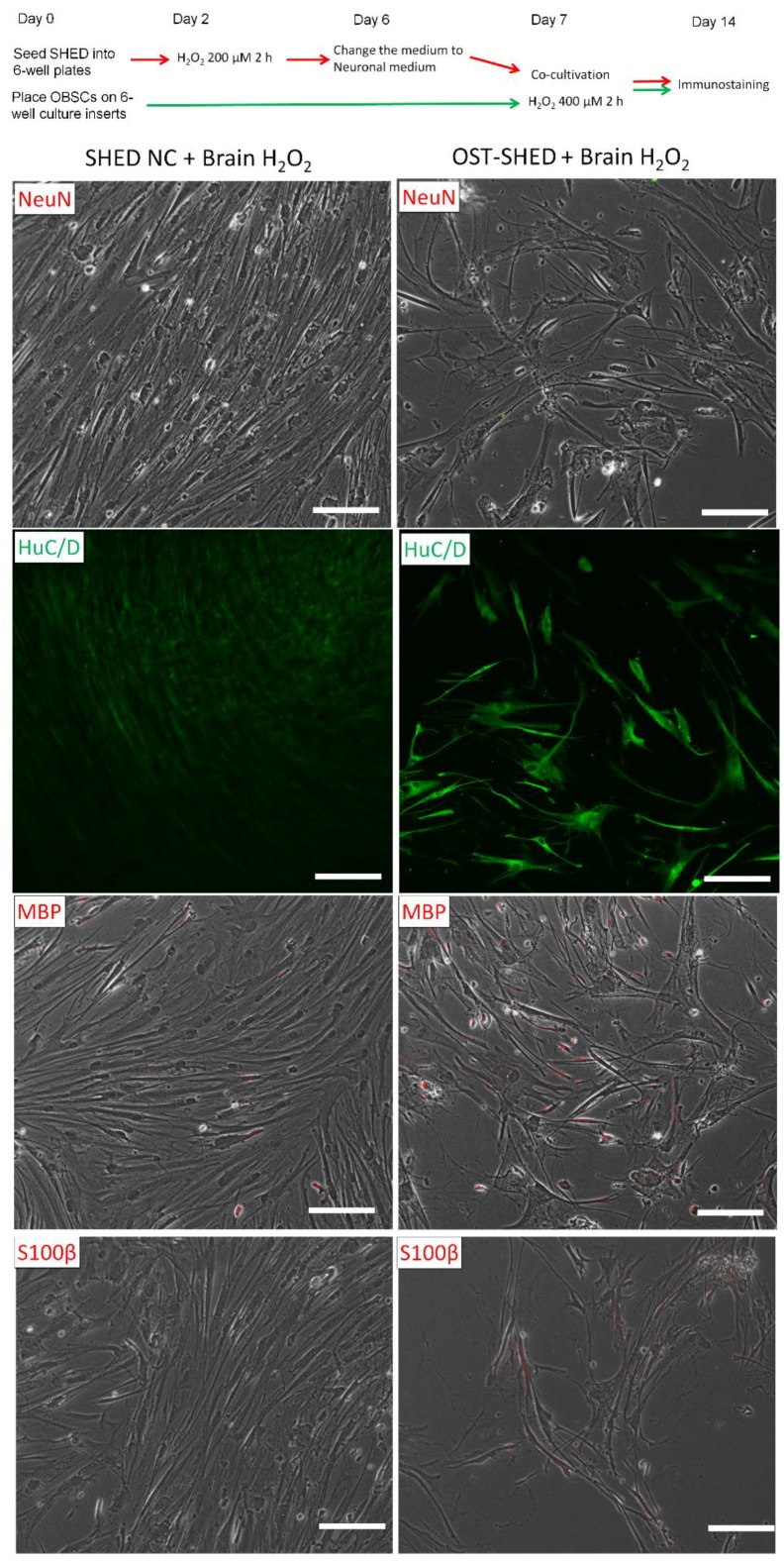
Auto-differentiation of OST-SHED. At the end of cultivation (day 14), co-cultivated SHED were subjected to immunostaining. The results showed that both normal SHED (SHED NC) and OST-SHED were negative to anti-NeuN antibody. SHED NC showed weak expression of HuC/D and MBP. S100β was absent in SHED NC. In contrast, OST-SHED showed much stronger expression of HuC/D, MBP and S100β.

We noticed that during co-cultivation with OBSCs, OST-SHED displayed the morphology of neuronal cells, such as long and branching dendrites. However, solo-cultivated OST-SHED did not display such neuronal morphology. We then checked the expression of neuronal markers, NeuN (marker for mature neurons), HuC/D (marker for neuronal cells), S100β (marker for astrocytes) and MBP (marker for oligodendrocytes) in co-cultivated SHED. As shown in Figure 4, normal SHED only showed weak expression of HuC/D. NeuN, MBP and S100β were hardly detected in normal SHED. In contrast, most of OST-SHED showed strong expression of HuC/D. MBP and S100β were expressed in about 50% of OST-SHED. However, OST-SHED did not express NeuN. These data suggest that co-cultivated OST-SHED could spontaneously differentiate into neuronal cells, especially glia cells, but not mature neurons.

### 2.5. Effect of SHED on H_2_O_2_-Induced Oxidative DNA Damage in OBSCs

**Figure 5:**
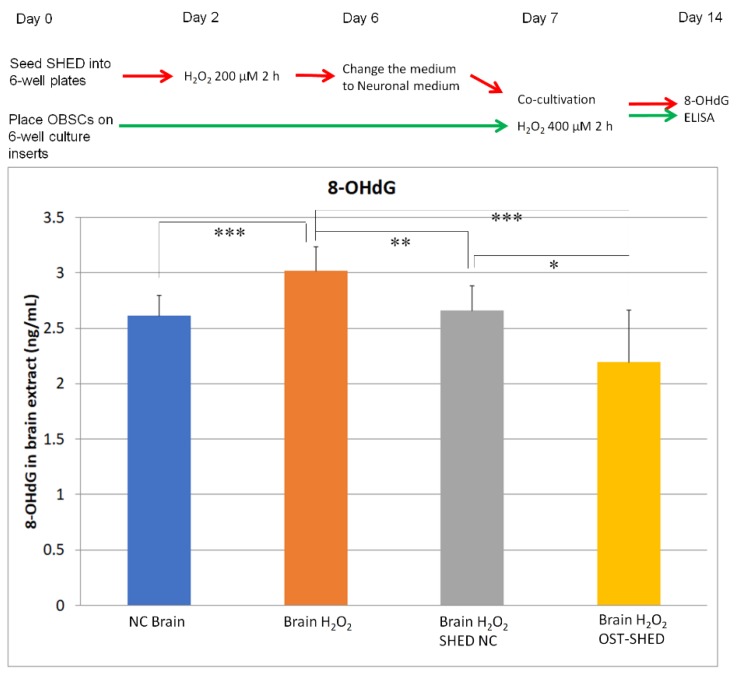
Cellular oxidative stress in OBSCs. At the end of cultivation (day 14), OBSCs were homogenized in RIPA buffer with ultrasound on ice. The tissue lysis was diluted and followed by 8-OHdG ELISA. Data are presented as mean ± SD. * *p* < 0.05, ** *p* < 0.01, *** *p* < 0.001.

H_2_O_2_ can induce oxidative DNA damage in cultivated cells [20], especially with copper and iron [21]. Since brain tissue contains high levels of copper and iron [22], H_2_O_2_ (400 μM) treatment must cause DNA damage in OBSCs. We therefore checked the concentration of 8-OHdG (8-hydroxy-2′-deoxyguanosine, the most commonly measured producer of oxidative DNA damage) in OBSCs’ extracts by ELISA. Figure 5 shows that H_2_O_2_ treatment markedly increased 8-OHdG production in solo-cultivated OBSCs (NC Brain v.s. Brain H_2_O_2_), but both normal and OST-SHED could significantly decrease 8-OHdG levels in co-cultivated OBSCs (*p* < 0.01, *p* < 0.001). In addition, OST-SHED showed remarkable superiority in usefulness to normal SHED (*p* < 0.05).

### 2.6. SHED Produce BDNF and May Stimulate IL-6 Secration in OBSCs

**Figure 6:**
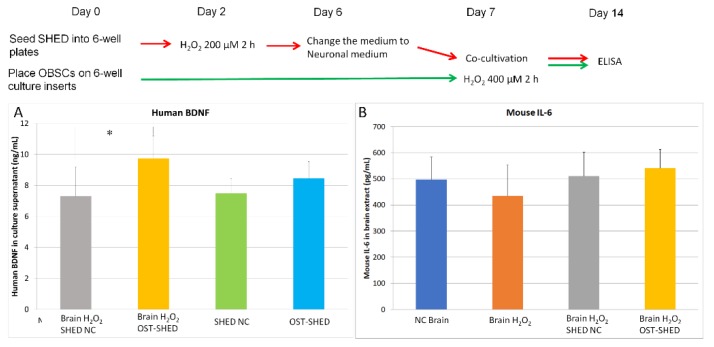
Production of human BDNF in SHED and mouse IL-6 in OBSCs. The culture supernatant was collected at the end of cultivation (day 14). (**A**), Levels of SHED-produced human BDNF detected by ELISA; (**B**), Levels of mouse brain tissue-produced IL-6 measured by ELISA. Data are presented as mean ± SD. * *p* < 0.05.

BDNF (brain-derived neurotrophic factor) is an important molecule for the survival of neurons [23]. We observed that all SHED (co-cultivated normal and OST- SHED, solo-cultivated normal and OST-SHED) produce certain levels of BDNF (7.3, 9.7, 7.5, 8.4 ng/mL, respectively). Co-cultivated OST-SHED produced more BDNF than normal SHED (Figure 6A) (*p* < 0.05). Although there are no statistical differences between solo- and co-cultivated OST-SHED, the data showed a tendency that co-cultivated OST-SHED produce more BDNF than solo-cultivated OST-SHED. These data suggest that SHED, especially OST-SHED, could promote neuronal survival and stimulate cell growth in co-cultivated OBSCs by providing neurotrophic support.

We also detected the release of inflammatory cytokine IL-6 in OBSCs. As shown in Figure 6B, although no statistical differences exist among the OBSCs (NC Brain, Brain H_2_O_2_, Brain H_2_O_2_ co-cultivated with SHED NC or OST-SHED), the trend suggests that H_2_O_2_ treatment decreases the secretion of IL-6, whereas normal SHED and OST-SHED increase IL-6 levels.

## 3. Discussion

It has been reported that H_2_O_2_ treatment (0.5–2.5 mM, 1 h) could induce massive cell death in hippocampal slice culture in vitro [16]. In the present study, our data showed that, H_2_O_2_ treatment (400 μM, 2 h) was able to significantly reduce cell viability in OBSCs (Figure 2F). As is well known, neurons, especially neurons in CA1 zone of the hippocampus, are vulnerable to oxidative stress [24]. However, Feeney et al. reported that glia cells are more vulnerable than neurons to H_2_O_2_-induced cell death [16]. Our immunostaining results showed that H_2_O_2_ treatment greatly decreased the number of mature neurons (NeuN positive cells) both in the CA1 and DG zones and reduced neural progenitors (PSA-NCAM positive cells) in the hippocampus (Figure 3) suggesting that H_2_O_2_ treatment mainly damaged neurons and progenitors. Although one week had passed after H_2_O_2_ treatment, the oxidative DNA damage was still significantly higher in H_2_O_2_-treated OBSCs than that in the negative control (Figure 5). These results suggest that H_2_O_2_ can induce severe damage in OBSCs through oxidative stress. Right after H_2_O_2_ treatment, we co-cultivated OBSCs with normal or OST-SHED. Both normal and OST-SHED produce high level BDNF (7.3–9.7 ng/mL) (Figure 6A), and they showed significant protection on oxidative injuries in OBSCs (Figure 2, Figure 3 and Figure 5). Similar to our findings, it has been reported that BDNF increases survival and neuronal differentiation of transplanted neuronal progenitors HNPCs [25]. Interestingly, although OST-SHED should have received H_2_O_2_-induced cellular damage (their proliferation rate was only half of normal SHED), they showed better protection and produced more BDNF than normal SHED in OBSCs.

As we mentioned in the introduction, H_2_O_2_ plays important roles on cellular signaling pathways. Brain stem cells-produced H_2_O_2_ can regulate intracellular growth signaling pathways and maintain normal cell proliferation through the enzyme NADPH oxidases 2 [26]. A recent report showed that H_2_O_2_ could induce chondrogenic differentiation of human adipose-derived stem cells [27]. Our data showed a similar result, that is, OST-SHED exhibited higher capacity than normal SHED for neuronal differentiation during co-cultivation with OBSCs. They were able to differentiate into glia cells (which are the main supporting cells for neurons [28]) (Figure 4). Compared to other ROS, H_2_O_2_ is a weaker oxidant. The effects of H_2_O_2_ to cells or tissues from useful to harmful depend on the dosage, treatment duration, and cell/tissue types. Park et al. treated neural stem cells (which were isolated from rodent embryonic brains) with H_2_O_2_ 200 μM for 24 h. They found that H_2_O_2_ treatment decreased cell viability to about 50% of non-treated cells [29]. We treated SHED with 200 μM H_2_O_2_ for 2 h and then further cultivated for 4–5 days. It is possible that short-term H_2_O_2_ treatment eradicated the weak cells, but the oxidative stress resistant cells survived and proliferated. These oxidative stress resistant cells have higher activity therefore they showed better protection for OBSCs.

IL-6 is known as an inflammatory cytokine which plays an essential role on inflammation and infection responses [30]. IL-6 also regulates the regeneration of liver cells [31] and airway ciliated cells [32]. In neuronal tissue, IL-6 is expressed in neuronal cells and behaves in a neurotrophin-like fashion to participate in neurogenesis [33]. Our results showed that, although there are no statistical differences, H_2_O_2_ treatment tended to decrease the secretion of IL-6 in OBSCs whereas SHED, especially OST-SHED, tended to increase IL-6 levels suggesting SHED is likely to stimulate OBSCs to secrete IL-6.

To our knowledge, this is the first report that oxidative stress-tolerant stem cells, OST-SHED, could produce neurotrophic factors, reduce neuronal cell death and support neural progenitors and these effects could be remarkably superior to normal SHED. Our findings may lead to the exploration for new therapeutic treatment for neurodegenerative diseases, strokes and traumatic brain injuries.

## 4. Materials and Methods

### 4.1. Cell Culture

To obtain stem cells from human exfoliated deciduous teeth (SHED), human deciduous incisors were obtained from a 7-year-old child at the Nippon Dental University Hospital at Tokyo under approved guidelines set by the Committee of Ethics, the Nippon Dental University School of Life Dentistry at Tokyo (authorization number: NDU-T2012-35, August 13, 2015) [19]. Similar to our previous reports, the incisors were washed three times in ice cold PBS (−) and then cut into halves from the tooth cervix with a diamond disc. Dental pulp tissues were carefully moved out from the pulp cavity. After washing three times with growth medium MEM-α (Thermo Fisher Scientific, Tokyo, Japan) containing 20% FBS, 100 units/mL penicillin, 10 mg/mL streptomycin and 1% Gibco^®^ GlutaMAX™ Supplement (Thermo Fisher Scientific), the dental pulp tissues were minced into 1- to 3-mm^2^ fragments, plated on 10-cm dishes with the growth medium, and cultured at 37 °C in a humidified tissue culture incubator with 5% CO_2_ and 95% O_2_. After 7–10 days of cultivation, the plastic-adherent confluent cells were treated with 0.05% trypsin containing 1 mM EDTA for 5 min to harvest pure mesenchymal cells. SHED were passaged and continuously subcultured and maintained in the complete growth medium. SHED from third to 14 passages were used in the experiments [19].

### 4.2. Organotypic Brain Slice Culture

Jcl:ICR mice (female, 3–4 weeks old) were purchased from CLEA Japan, Inc. (Tokyo, Japan). 22 mice were used in this study. The animal experiments were performed under approved guidelines set by the Animal Ethics Committees, the Nippon Dental University School of Life Dentistry at Tokyo (authorization number: 16-02-2, May 8th 2018). To prepare the brain slices, we used sharp utility scissors to cut the head of the mouse and scooped out the brain quickly with a rounded spoon micro spatula and placed it into the ice cold neural medium (Neurobasal^®^ Medium, Thermo Fisher Scientific). We then verticality cut the brain hemisphere to slices with a thickness of 200 µM with a vibratome (NeoLinearSlicer MT, DOSAKA EM CO., LTD. Kyoto, Japan). The brain slices with dentate gyrus of the hippocampus (8 per brain) were then carefully cut into half from the middle line and gently transferred onto six-well culture inserts (PICM0RG50, Millipore, Billerica, MA, USA, Tokyo, Japan) which were coated with 200 µL matrigel (354234, Corning, New York, NY, USA, Tokyo, Japan). The brain slices were cultivated in 900 µL neural medium containing B-27 Supplement (Thermo Fisher Scientific)) at 35 °C in a humidified tissue culture incubator with 5% CO_2_. The experiments were carried out after 7 days in vitro. For co-cultivation, SHED (4 × 10^4^ cells/well) were seeded into a six-well plate and then co-cultivated with brain slices as shown in Figure 1.

### 4.3. Hydrogen Peroxide Treatment

30% hydrogen peroxide (H_2_O_2_) (081-04215, FUJIFILIM Wako Pure Chemical Co., Tokyo, Japan) was diluted first in double distilled water then culture medium just prior to each experiment. SHED (4 × 10^4^ /well) were seeded in 6-well plates and cultivated over 36 h. Then, SHED were incubated with 200 μM H_2_O_2_ at 37 °C in a humidified incubator with 5% CO_2_ for 2 h. After 2 h of exposure, cells were rinsed and further cultivated in normal culture medium without H_2_O_2_ for another 4–5 days. Control cells were similarly treated without H_2_O_2_. For H_2_O_2_ treatment to OBSCs, OBSCs were incubated with 400 μM H_2_O_2_ at 35 °C in a humidified incubator with 5% CO_2_ for 2 h. Control OBSCs were similarly exposed to the culture medium without H_2_O_2_. After 2 h of exposure, OBSCs were rinsed and cultivated in normal culture medium without H_2_O_2_ (Figure 1).

### 4.4. Enzyme-Linked Immunosorbent Assay (ELISA)

To determine levels of 8-OHdG, BDNF and IL-6 in the slice culture medium and the extracts of OBSCs, samples were analyzed using commercially available ELISA kits (New 8-OHdG Check ELISA kit, JaICA Co. Ltd. (Shizuoka, Japan), Human BDNF ELISA kit, RAB0026, Sigma-Aldrich, Mouse IL-6 ELISA Kit, RAB0308-1KT, Sigma-Aldrich, Tokyo, Japan). Culture medium was obtained during and after the cultivation of OBSCs. At the end of cultivation, OBSCs (2 slices) from a single culture membrane were carefully removed from the culture insert without containing matrigel. The OBSCs were then homogenized in 100 μL RIPA lysis buffer (WSE-7420 EzRIPA Lysis kit, ATTO Co., Tokyo, Japan) to obtain the extracts. Protein concentrations of the extracts were measured with a protein quantification kit (Protein Quantification Kit-Rapid, Dojindo Molecular Technologies, Inc., Kumamoto, Japan). For each ELISA, culture medium or OBSCs extracts (with same concentration of protein) were diluted to bring the expected concentration within the range of the standard curve and reacted with first and secondary antibodies, streptavidin-HRP and detection solution according to the manufacturer’s instruction. The reaction was stopped using the stop solution (from the ELISA kits) and absorbance read at 450 nm using a microplate reader (SH-9000Lab, Hitachi, Tokyo, Japan).

### 4.5. Immunohistochemistry

At the end of cultivation, SHED and OBSCs were fixed in 3 mL 4% paraformaldehyde at 4 °C overnight. The samples were incubated with serum blocking solution for 60 min to suppress the non-specific binding of IgG, and then incubated with saturating levels of primary antibodies for 2 days at 4 °C. The primary antibodies used were anti-NeuN (Abcam, ab177487), anti-HuC/D (Thermo Fisher Scientific, A-21271), anti-MBP (Abcam, ab216668), anti-S100β (Abcam, ab52642) and anti-PSA-NCAM (Thermo Fisher Scientific, 14-9118-80). After carefully washing with 1% Triton in PBS (−), specimens were reacted with fluorochrome-conjugated secondary antibody (Thermo Fisher Scientific, A11001, A11012) diluted to 2 µg/mL in 1% Triton in PBS (−) with 1.5% normal blocking serum for 3 days at 4 °C in dark. The nuclei were stained with DAPI. Samples were imaged and analyzed with a confocal laser scanning microscopy (LSM700, Carl Zeiss Microscopy Co., Ltd., Tokyo, Japan). The fluorescence density was quantified by Image J software [34].

### 4.6. Cell Viability Assay

Cell viability in cultivated brain slices were measured by PrestoBlue^®^ Assay according to the standard protocol. Brain slices were cultivated on six-well culture inserts (3 slices/insert) as we described in Section 4.2. The culture inserts were then transferred to a new six-well plate and incubated for 3 h at 35 °C with fresh neural medium (900 µL) supplemented with 10 vol.% PrestoBlue^®^ (Thermo Fisher Scientific, A13261). The PrestoBlue^®^ reduction by the cells in the brain slices expressed as fluorescence intensity units was measured on a microplate reader (SH-9000Lab, Hitachi, Tokyo, Japan) with excitation 560 nm and emission 590 nm [19].

### 4.7. Statistical Analysis

Statistical analysis was carried out similar to in our previous report; all data, expressed as mean ± SD, were processed statistically by GNU PSPP Statistical Analysis Software (version 0.8.2-gad9374) and the OpenStat program by Bill Miller. A one-way analysis of variance followed by least significant difference test (equal variances assumed) or the Dunnett’s T3 test (equal variances not assumed) was used for statistical analysis. The differences of the data were considered when *p* < 0.05 [19]. All experiments were repeated 3–5 times independently.

## Abbreviations

IBD	ischemic brain damage
TBI	traumatic brain injury
OBSCs	organotypic brain slice cultures
SHED	stem cells from human exfoliated deciduous teeth
OST-SHED	oxidative stress-tolerant stem cells from human exfoliated deciduous teeth
ROS	reactive oxygen species
DG	dentate gyrus
CNS	central nervous system
PSA-NCAM	polysialylated-neural cell adhesion molecule
